# Multifaceted Roles of Vitamin D for Diabetes: From Immunomodulatory Functions to Metabolic Regulations

**DOI:** 10.3390/nu16183185

**Published:** 2024-09-20

**Authors:** Chan Yoon Park, Sunhye Shin, Sung Nim Han

**Affiliations:** 1 Department of Food & Nutrition, College of Life Care Science Technology, The University of Suwon, Hwaseong-si 18323, Republic of Korea; cypark@suwon.ac.kr; 2 Department of Food and Nutrition, College of Science and Convergence Technology, Seoul Women’s University, Seoul 01797, Republic of Korea; sshin@swu.ac.kr; 3 Department of Food and Nutrition, College of Human Ecology, Seoul National University, Seoul 08826, Republic of Korea; 4 Research Institute of Human Ecology, Seoul National University, Seoul 08826, Republic of Korea

**Keywords:** vitamin D, diabetes, innate immunity, inflammation, β-cell function

## Abstract

Numerous studies have established associations between vitamin D and diabetes. The vitamin D receptor is widely distributed throughout the human body, including in pancreatic beta cells (β-cells), hepatocytes, and immune cells. Therefore, vitamin D’s effect on the risk, progression, or complications of diabetes may be mediated through various mechanisms. These include the regulation of insulin secretion or sensitivity and modulation of β-cell function and its immunomodulatory and anti-inflammatory effects. This review extensively explores the relationship between vitamin D status and diabetes, as well as the preventive or therapeutic effects of vitamin D supplementation on diabetes from human studies. Additionally, it examines in detail the impact of vitamin D on immune and inflammatory responses in the diabetic milieux and β-cell function to better understand the underlying mechanisms through which vitamin D influences diabetes.

## 1. Introduction

Vitamin D is a fat-soluble vitamin that can be derived from a few dietary sources. It exists in two forms: vitamin D_3_ (cholecalciferol), the mammalian form, and vitamin D_2_ (ergocalciferol), the plant or fungal form. Structurally, vitamin D is a secosteroid hormone that can be endogenously synthesized from 7-dehydrocholesterol in the skin via ultraviolet (UV) exposure. In the human body, endogenous or dietary vitamin D is initially metabolized to 25-hydroxyvitamin D (25(OH)D) by 25-hydroxylases (CYP2R1 and CYP27A1) in the liver and, subsequently, to the biologically active form 1,25-dihydroxyvitamin D (1,25(OH)_2_D) by 1-hydroxylase (CYP27B1), mainly in kidney. Since 25(OH)D is the major circulating form of vitamin D and remains stable by exhibiting a half-life of approximately 15–20 days, total serum 25(OH)D concentration is currently considered the best indicator of vitamin D status [1]. A 25(OH)D level <20 ng/mL (50 nmol/L) defines vitamin D deficiency, whereas that >30 ng/mL (75 nmol/L) indicates vitamin D sufficiency [1]. After 1-hydroxylation, active 1,25(OH)_2_D regulates target gene transcription by binding to the nuclear vitamin D receptor (VDR) and retinoid X receptor (RXR) and is subsequently translocated into the nucleus [2]. In addition to the genomic responses of vitamin D–VDR, vitamin D initiates rapid cellular nongenomic responses through a putative plasma-membrane-associated VDR or protein disulfide isomerase A3 (PDIA3), which has been found to stimulate the release of secondary messengers and modulate various intracellular processes [3]. Owing to the wide distribution of VDRs throughout the human body, including pancreatic beta cells (β-cells), hepatocytes, and immune cells, numerous studies have established associations between vitamin D and diabetes. During the past decade, vitamin D has demonstrated potential benefits beyond bone health and calcium homeostasis in various diseases, including type 2 diabetes [4,5,6]. Vitamin D has been implicated in anti-diabetic activity as it regulates insulin secretion or sensitivity, modulates β-cell function, exerts anti-inflammatory effects, and alerts the immune system [6]. Diabetes is a metabolic disease characterized by high blood glucose caused by islet dysfunction and insulin resistance. The prevalence of type 2 diabetes mellitus (T2DM) is generally high in older adults and characterized by increased insulin resistance. Type 1 diabetes mellitus (T1DM) is an autoimmune disease characterized by the destruction of pancreatic β-cells owing to interactions between susceptibility genes and environmental factors [7]. Multiple human studies have investigated the relationship between vitamin D deficiency and the incidence of both types of diabetes.

## 2. Vitamin D Status and Diabetes (Human Studies)

### 2.1. Vitamin D Deficiency and T2DM

Numerous observational studies, including both longitudinal and cross-sectional studies, have consistently reported that vitamin D deficiency, characterized by a low level of serum 25(OH)D, is associated with an increased risk of T2DM. Several large cross-sectional studies and case–control studies conducted in various populations [8,9,10,11,12,13,14,15] have revealed significant inverse associations between circulating 25(OH)D levels and T2DM. For instance, a cross-sectional study involving 6228 participants from the National Health and Nutrition Examination Survey (NHANES) found vitamin D status to be negatively correlated with diabetes in non-Hispanic Whites and Mexican Americans, but not in non-Hispanic Blacks, after adjusting for sex, age, body mass index, physical activity, and seasonality [8]. Furthermore, plasma 25(OH)D concentration was positively correlated with insulin sensitivity and pancreatic β-cell functions in a cross-sectional study involving healthy populations [16].

Additionally, numerous longitudinal studies have reported similar findings [12,17,18,19]. A large recent prospective cohort study of adults (n = 5272 for wave 1; n = 3828 for wave 3) showed that baseline vitamin D status was not only cross-sectionally associated with an increased likelihood of prevalent diabetes, but longitudinal analyses after a 4-year follow-up also showed that participants with vitamin D deficiency had a 62% higher likelihood of developing prediabetes (Relative Risk Ratio: 1.62 (1.12–2.35)) [12]. A prospective cohort study involving 9841 participants, of whom 810 developed T2DM over 29 years of follow-up, found that lower baseline 25(OH)D concentrations were associated with a higher cumulative incidence of T2DM [17]. Another 10-year cohort study involving 524 participants without diabetes from the Ely Study identified inverse associations between baseline serum 25(OH)D levels and the 10-year risk of hyperglycemia and insulin resistance [19]. A pooled analysis of two nested case–control studies comprising men and women aged 40–74 years yielded relative odds of T2DM (between the highest and lowest serum 25(OH)D quartiles) of 0.28 (0.10–0.81) and 1.14 (0.60–2.17) in men and women, respectively, after adjustment [18]. However, several studies involving older women (mean age: 66.3 years) or older participants (51% women; mean age: 67.9 years) did not establish a significant association between baseline 25(OH)D levels and the incidence of diabetes [20,21]. Despite this, meta-analyses by Song et al. [22], who analyzed 21 prospective studies with 76,220 participants and 4996 incident cases of T2DM, and Khan et al. [23], who assessed 18 prospective studies with 210,107 participants and 15,000 metabolic events, both demonstrated a significantly inverse association between circulating 25(OH)D levels and T2DM. Also, a recent meta-analysis of participants in European descent studies (n = 120,618) found that a 1-standard-deviation-higher level of total 25(OH)D was associated with 20% lower risk of T2DM [24]. These findings reinforce the link between vitamin D status and the incidence of T2DM.

### 2.2. Vitamin D Deficiency and T1DM

Several human studies have reported that people with T1DM have relatively low circulating vitamin D levels [25,26,27,28,29] (Table 1). Epidemiological research indicates a potential link between early-life vitamin D deficiency and the later onset of T1DM [30]. This is supported by previous findings wherein comparatively high T1DM incidence rates were noted in high-latitude regions with low UVB irradiance, such as Finland, Sweden, and Norway, as well as a seasonal pattern of T1DM onset, with peaks in winter, early spring, and late autumn [31,32,33].

An age-, sex-, and ethnicity-matched case–control study (n = 170 in each group) revealed that vitamin D deficiency was considerably more prevalent in children with T1DM than in those without [25]. Additionally, both 25(OH)D and 1,25(OH)_2_D levels were significantly lower in newly diagnosed patients with T1DM than in healthy sex- and age-matched controls [26]. However, whether these levels were already lower prior to diagnosis remains uncertain. 

While certain studies have suggested a link between vitamin D concentration and β-cell autoantibody status in children at risk of T1DM, the evidence remains inconclusive. For example, a cross-sectional analysis found 25(OH)D levels to be lower in children with multiple islet autoantibodies than in those without [34]. However, another study reported no association between circulating vitamin D status and β-cell autoantibody positivity [35]. Furthermore, a prospective study involving 1316 young people with autoantibody-positive T1DM revealed an inverse association between vitamin D level and fasting C-peptide, which is synthesized when insulin is produced and released, over a 2-year follow-up period [36]. Norris et al. [37] suggested that these conflicting results potentially emanate from the failure to account for genetic variations in the vitamin D pathway. Their findings indicated an association between higher childhood 25(OH)D levels and a lower risk of islet autoimmunity, with this association being influenced by the VDR rs7975232 polymorphism. This suggests that both vitamin D and VDR potentially play a role in the development of islet autoimmunity in children carrying a greater genetic risk of T1DM.

### 2.3. Vitamin D Supplementation and Diabetes

Although numerous observational studies have found low serum 25(OH)D levels to be associated with an increased prevalence of both T1DM and T2DM as well as elevated blood glucose levels, whether these associations are causal remains unclear [24]. The hypothesis that vitamin D supplementation can reduce diabetes risk is biologically plausible because vitamin D regulates insulin secretion from the pancreas by enhancing β-cell function and inhibits insulin resistance by mitigating chronic inflammation [38]. Nevertheless, the effects of vitamin D supplementation on glycemic control are inconsistent. Methodological limitations, such as variations in vitamin D dosage, duration, and form, along with individual differences, such as baseline serum 25(OH)D levels, responsiveness to vitamin D, and genetic variants influencing vitamin D metabolism, potentially contribute to these discrepancies [22,39,40,41]. Additionally, the effectiveness of vitamin D supplementation may vary depending on health status (diabetic or non-diabetic), diabetes type (type 1 or type 2), and other outcome variables (e.g., diabetes prevalence, blood glucose concentration, and insulin level).

#### 2.3.1. Effects of Vitamin D Supplementation on T2DM Prevention

Several randomized controlled trials (RCTs) involving more than 500 participants and lasting >2 years were conducted to determine whether vitamin D supplementation lowers the risk of T2DM in individuals with prediabetes [41,42,43,44] (Table 2). Despite the lack of statistically significant differences, the supplemented groups generally exhibited a lower risk of diabetes than the placebo groups, despite variations in the quantity, form, and duration of vitamin D intake as well as differences in baseline vitamin D levels. For instance, in an RCT involving 2423 participants with prediabetes (fasting plasma glucose: 100–125 mg/mL), oral vitamin D intake (cholecalciferol; 4000 IU/day) for a median duration of 2.5 years did not significantly affect the hazard ratio for developing diabetes compared with placebo intake [41]. Similarly, Jorde et al. [42] found that 5-year vitamin D supplementation (20,000 IU per week) did not effectively prevent progression from prediabetes to diabetes, possibly because the participants had not been screened for vitamin D insufficiency at baseline. Additionally, the Diabetes Prevention with Active Vitamin D (DPVD) study, which was conducted in Japan and involved 1256 participants, revealed that supplementation with eldecalcitol (an analog of active 1,25(OH)_2_D) did not significantly reduce diabetes incidence among individuals with prediabetes [44].

In contrast, a few short-term or smaller clinical studies (n <100) that did not assess T2DM incidence but focused on related indicators, such as insulin secretion and glycated hemoglobin (HbA1c), have suggested the beneficial effects of vitamin D supplementation [45,46,47,48,64]. A 16-week controlled study reported that 2000 IU/day of cholecalciferol improved pancreatic β-cell function, as indicated by an enhanced disposition index and insulin secretion [45]. Moreover, cholecalciferol (5000 IU/day) supplementation for 6 months significantly increased peripheral insulin sensitivity and β-cell function [46].

Several meta-analyses of RCTs or prospective cohort studies examining the impact of vitamin D supplementation on diabetes prevention have yielded conflicting results [65,66,67,68]. Systematic reviews of 35 trials involving 43,407 adults with normal glucose tolerance, prediabetes, or T2DM found no significant effects of vitamin D on insulin resistance, insulin secretion, or HbA1c levels compared to controls [65]. Similarly, a meta-analysis of prospective cohort studies with 187,582 participants and 9456 incident cases also established no association between vitamin D intake and T2DM incidence [67]. However, a meta-analysis of 47 RCTs involving 44,161 adults without diabetes (median duration: 4 months, dose: 4000 IU/day) revealed that vitamin D supplementation significantly reduced fasting blood glucose, fasting insulin, and homeostasis-model-assessment-estimated insulin resistance (HOMA-IR) levels, though no differences were observed in insulin secretion or β-cell function indexes [66]. These studies suggest that, while vitamin D supplementation may improve T2DM-related indicators in individuals with prediabetes, it does not demonstrate a significant association with the incidence of T2DM; thus, further research is warranted.

#### 2.3.2. Effects of Vitamin D Supplementation on Patients with T2DM

Several recent meta-analyses of RCTs have assessed the impact of vitamin D supplementation on glucose and insulin regulation in individuals with existing T2DM [49,50,51] (Table 2). These studies concluded that vitamin D supplementation can improve glycemic control outcomes, such as fasting plasma glucose and HOMA-IR levels, by increasing 25(OH)D concentration across the entire cohort. Li et al. [50] discovered that these effects were particularly prominent in subgroups receiving short-term, high-dose vitamin D as well as among Middle Eastern participants and those with baseline vitamin D deficiency, with a decrease in fasting plasma glucose levels being shown. Similarly, a meta-analysis including 46 RCTs reported significant and notable improvements in fasting plasma glucose, HOMA-IR, and HbA1c levels in the vitamin-D-supplemented group with vitamin D deficiency [49]. These findings suggest that vitamin D supplementation potentially improves glycemic control in patients with T2DM, particularly those with vitamin D insufficiency.

#### 2.3.3. Effects of Vitamin D Supplementation on Patients with T1DM

Multiple studies have explored the therapeutic effects of vitamin D supplementation on individuals with T1DM as well as its potential preventive effects against T1DM onset (Table 2). Despite the challenges associated with conducting clinical trials targeting high-risk groups for T1DM, maternal birth cohort studies involving children have been conducted to assess the effect of vitamin D on T1DM prevention [52,53,69].

In early childhood, vitamin D supplementation was related to a lower risk of T1DM development [53,54,69], whereas a lack of supplementation was associated with a higher risk [70]. Maternal consumption of cod liver oil, a commonly used dietary vitamin D supplement for infants, during pregnancy was inversely related to the incidence of T1DM in their children, and maternal vitamin D supplementation was linked to a reduced risk of islet autoimmunity in offspring [52,55]. Corroborating these findings, another birth cohort involving 12,055 pregnant women reported that regular vitamin D supplementation in the first year of life was associated with a reduced incidence of T1DM (rate ratio [RR] = 0.12, 0.03–0.51) compared with no supplementation, after adjusting for neonatal, anthropometric, and social characteristics [53]. Specifically, children who regularly received the recommended 2000 IU/day of vitamin D had an RR value of 0.22 (0.05–0.89) compared with those receiving less than the recommended amount. These results suggest that adequate vitamin D supplementation in infants may offer protective benefits against T1DM. A meta-analysis incorporating data from four case–control studies and one cohort study examined whether vitamin D supplementation in infants alleviated the risk of T1DM [69]. The study found that children receiving vitamin D supplementation had a 29% reduced risk of developing T1DM, indicating vitamin D’s potential protective role in β-cells and the immune system.

Additionally, investigations into the therapeutic effects of vitamin D supplementation on the progression and management of established T1DM have yielded similar results [56,57,58,71] (Table 2). A meta-analysis of RCTs, including seven studies with 287 participants, indicated that vitamin D supplementation—specifically in the forms of alfacalcidol and cholecalciferol—resulted in reduced insulin doses as well as maintained or increased fasting/stimulated C-peptide levels, suggesting a beneficial effect in the management of T1DM [71]. However, calcitriol (1,25(OH)_2_D), the active form of vitamin D, did not significantly affect T1DM-related factors, such as C-peptide level and daily insulin doses [59,60,61]. Conversely, Pitocco et al. [56] revealed that 1-year supplementation with 1,25(OH)_2_D (0.25 µg/48 h) reduced insulin doses at 3 and 6 months but did not affect C-peptide or HbA1c levels in patients with T1DM.

However, RCTs investigating the effect of cholecalciferol on T1DM progression have demonstrated therapeutic benefits [57,62,63]. For example, a study involving 53 children with both T1DM and vitamin D deficiency found that supplementation with cholecalciferol (300,000 IU) and calcium (40 mg/kg/day) led to a reduction in HbA1c levels [63]. Additionally, Gabbay et al. [57] reported that cholecalciferol supplementation (2000 IU/day for 18 months) resulted in increased monocyte chemoattractant protein-1 (MCP-1) levels, a higher percentage of regulatory T cells (Tregs), and lower cumulative incidence of progression to undetectable (≤0.1 ng/mL) fasting and stimulated serum C-peptide in patients with new-onset T1DM. These findings suggest that vitamin D may have both preventive and therapeutic effects on T1DM. However, its efficacy appears to vary depending on the form of vitamin D used, highlighting the need for further research.

Various studies have proposed that polymorphisms in the *VDR* gene are associated with susceptibility to T1DM. Furthermore, several meta-analyses investigating the association between *VDR* gene polymorphisms (FokI, ApaI, TaqI, and BsmI) and T1DM have generated controversial results [72,73,74,75]. A meta-analysis including 57 case–control studies from 26 published studies found BsmI polymorphism to be associated with an increased risk of T1DM, especially in Asian populations [73]. Similarly, Sahin et al. [72] reported that BsmIBB, BsmIBb, and TaqItt polymorphisms were associated with an increased risk of T1DM in children based on a meta-analysis of nine studies involving 1053 patients and 1017 controls. In contrast, two other meta-analyses identified no significant association between *VDR* gene polymorphisms and T1DM risk [74,75]. A recent meta-analysis, which included 39 studies published in 2020, found no significant association between *VDR* gene polymorphisms and T1DM risk [74]. However, subgroup analyses revealed significant associations between FokI and BsmI polymorphisms and T1DM, with FokI exhibiting a negative association in Africans and BsmI displaying a positive association in Americans. Zhai et al. [74] suggested that these inconsistencies potentially result from variations in detection methods and diagnostic criteria, clinical heterogeneity, small sample sizes, and the interaction between genetic and environmental factors.

## 3. Role of Vitamin D in Immune Functions and Inflammatory Responses in Diabetes

### 3.1. Alteration of Immune Responses by Diabetes

The alteration of immune responses by diabetes is evidenced by the increased risk of infection in persons with diabetes. In a retrospective cohort study, 102,493 patients with diabetes aged 40–89 years were matched with 213,518 control participants without diabetes and compared for 19 infection categories over a 7-year period [76]. People with diabetes had higher rates for all infections. The incidence rate ratios (IRRs) for infection-related hospitalization were 3.71 for T1DM and 1.88 for T2DM, and those for infection-related mortality were 7.72 for T1DM and 1.92 for T2DM. Using data from the National Health Insurance Service National Sample Cohort, Kim et al. [77] compared 17 infection categories between 66,426 participants with diabetes and 132,852 non-diabetes controls over a 9-year period. The diabetes group exhibited a higher risk of infection, except infective otitis externa, than the control group. The adjusted IRRs (aIRRs; adjusted for household income, hypertension, dyslipidemia, cardiovascular disease, and chronic disease) were 1.76, 1.83, and 2.21 for respiratory infection, urinary tract infection, and sepsis-related hospitalization, respectively. Hepatic abscess (10.17), central nervous system infections (8.72), and skin and soft-tissue infections other than cellulitis (3.52) yielded the highest aIRRs.

The alteration of both innate and adaptive immunities as well as hyperinflammation have been reported in diabetes [78]. Innate immune response changes observed in diabetes include attenuated neutrophil chemotaxis and phagocytosis, natural killer (NK) cell dysfunction, and impaired dendritic cell (DC) function. NK cells from patients with T2DM had significantly lower subsets positive for activating receptors NK group 2, member D (NKG2D), and NK cell p46-related protein (NKp46) [79]. A significantly inverse correlation between NKG2D-expressing cells and HbA1c concentration was observed, indicating that hyperglycemia plays a role in NK cell dysfunction. In contrast, in a systematic review and meta-analysis of clinical studies which included 13 studies reporting on 491 adult patients with T2D and 1064 nondiabetic controls, the pooled effect estimates showed increased levels of NK cells in patients with T2DM compared to controls, suggesting that NK cells were activated. However, the authors concluded that the changes in NK cell counts in patients with T2DM remain unclear [80].

High glucose concentration is also a critical regulator of lung DC function as the expression of co-stimulatory molecules and antigen transport are impaired by hyperglycemia [81]. DCs potentially play important roles in adipose tissue inflammation and diabetic nephropathy, and the balance between tolerogenic and pro-inflammatory functions is critical for the regulation of inflammation and progression of kidney injury [82,83]. Therefore, altered DC function owing to diabetes not only affects antigen presentation and T cell priming but also aggravates complications associated with diabetes.

### 3.2. Effects of Vitamin D on Immune Functions in Diabetes

Vitamin D possesses immunomodulatory functions affecting both the innate and adaptive immune systems [84,85]. However, studies on the immunological effects of vitamin D in diabetic milieux are limited. Eight-week vitamin D supplementation (10,000 IU/kg diet) was shown to elicit higher NK cell activity in diabetic mice [86]. NK cell activity in the diabetic mice was approximately 70% lower than that in the control mice. The percentage of transforming growth factor-beta (TGF-β)-positive splenocytes was higher in diabetic mice and tended to be lower in their vitamin-D-supplemented counterparts. TGF-β inhibits the activation and functions of NK cells by suppressing the induction of mTOR activity mediated by IL-2 or IL-15 [87]. NK cell subset analysis indicated that vitamin-D-supplemented animals had a higher percentage of CD11b single-positive NK cells, which are in mature stage 3, and revealed a positive correlation between NK cell activity and CD11b single-positive NK cells [86]. Vitamin D supplementation also increased the production of interleukin (IL)-12, which is responsible for enhancing NK-cell-mediated cytotoxicity [88] and Bcl2 and Tbx21 expression. Bcl-2 inhibits NK cell apoptosis, and T-bet is required for the final maturation of NK cells [89,90]. Vitamin D has also been reported to have stimulatory effects on innate immunity, such as increasing the production of IL-1 and bactericidal peptide by monocytes and macrophages [84]. Therefore, vitamin D may provide protection against infection in diabetic conditions by enhancing the first line of defense through NK cells and macrophages.

In contrast, vitamin D primarily exerts inhibitory effects on adaptive immunity. This may work to regulate the autoimmune characteristics of T1DM. Non-obese diabetic (NOD) mouse supplementation with vitamin D (2200 IU/kg diet for 28 days) downregulated cathepsin G expression and inhibited CD4+ T cell activation in the pancreatic β-cell immune environment [91]. Oral vitamin D analog (0.1 μg/kg) treatment five times per week for 8 weeks decreased the production of chemokines chemokine (C–X–C motif) ligand (CXCL)-10, C–C motif chemokine ligand (CCL)-2, and CCL5 by islet cells, and blocked Th1 cell infiltration into the pancreas in NOD mice [92].

### 3.3. Effects of Vitamin D on Inflammatory Responses in Diabetes

Diabetes reportedly increases reactive oxygen species (ROS) levels and induces chronic low-grade inflammation, thereby contributing to diabetes-related comorbidities [93]. Vitamin D potentially improves diabetes by exerting anti-inflammatory effects. In an RCT, improving vitamin D status in patients with T2DM via 12-week vitamin D supplementation (1000 IU vitamin D3/day) resulted in decreased levels of the inflammatory markers high-sensitivity C-reactive protein (hs-CRP), serum amyloid A, and IL-6 [94]. Johy et al. [95] reported that patients with T2DM who had undergone 6 months of vitamin D supplementation (60,000 IU cholecalciferol/wk for the first 3 months followed by 60,000 IU/month for the subsequent 3 months) displayed reduced serum levels of IL-18, tumor necrosis factor-alpha (TNF-α), interferon-gamma (IFN-γ), CXCL10, CXCL12, CCL2, CCL5, CCL11, and platelet factor 4 compared with those at baseline. Twelve-week vitamin D intervention (50,000 IU every 2 weeks) in patients with diabetic hemodialysis led to significant reductions in serum hs-CRP and plasma malondialdehyde MDA and an increase in plasma total antioxidant capacity [96].

Vitamin D’s anti-inflammatory effects have been observed in different tissues implicated in diabetic complications, such as renal and retinal tissues. The suggested mechanisms underlying vitamin D’s anti-inflammatory action include the suppression of the Akt/NF-κB/COX-2 pathway in macrophages and downregulation of hyperglycemia-induced intracellular ROS production in human retinal microvascular endothelial cells (HRMECs) [97,98]. Reduced ROS production resulted in inhibition of the TRX-interacting protein (TXNIP)/NOD-like receptor family and pyrin domain-containing 3 (NLRP3) inflammasome pathway activation [98]. In addition, eight-week vitamin D treatment (3500 IU/kg/week) alleviated diabetic peripheral neuropathy in rats by suppressing Notch1 and Wnt-10α/β-catenin signaling and by reducing inflammation in the nerve [99].

**Figure 1:**
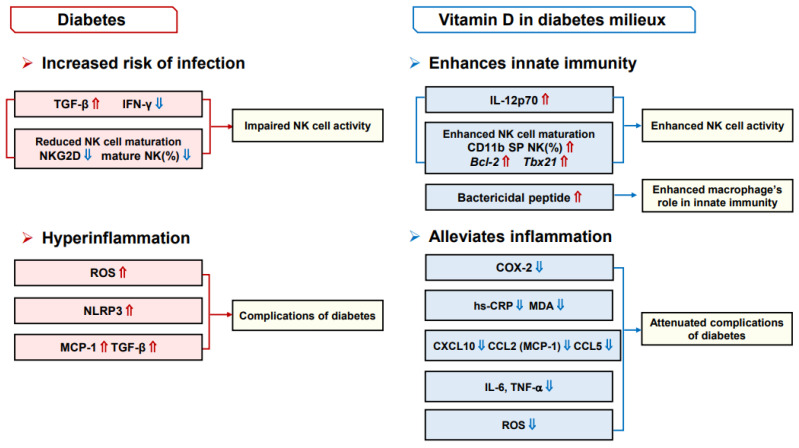
Effects of diabetes and vitamin D on immune and inflammatory responses. Impaired innate immunity observed with diabetes leads to increased risk of infection and hyperinflammation can aggravate the complications of diabetes. Vitamin D can enhance NK activity and bactericidal activity of macrophages. Anti-inflammatory effects of vitamin D can alleviate complications of diabetes. Abbreviations: ⇑, increase; ⇓, decrease; TGF-β, transforming growth factor-beta; IFN-γ, interferon-gamma; NK cell, natural killer cell; NKG2D, activating receptors NK group 2, member D; ROS, reactive oxygen species; NLRP3, NOD-like receptor family and pyrin domain-containing 3; MCP-1, monocyte chemoattractant protein-1; IL-12p70, interleukin-12p70; CD11b, cluster of differentiation 11b; *Bcl-2*, B-cell lymphoma *2*; *Tbx21*, T-box transcription factor 21; COX-2, cyclooxygenase-2; hs-CRP, high-sensitivity C-reactive protein; MDA, malondialdehyde; TNF-α, tumor necrosis factor-alpha; CXCL10, chemokine (C–X–C motif) ligand (CXCL)-10; CCL2, C–C motif chemokine ligand (CCL)-2.

Effects of diabetes and vitamin D on immune and inflammatory responses are depicted in Figure 1.

## 4. Effects of Vitamin D on Pancreatic β-Cell Function and Insulin Release in Diabetes

Vitamin D plays a significant role in immune system development in early life [100,101,102]. Vitamin D deficiency potentially increases the risk of autoimmunity [103,104,105], whereas vitamin D sufficiency can prevent the pathogenesis of autoimmune diseases, including T1DM [106,107,108].

### 4.1. Mechanisms

In T1DM, pancreatic β-cell-specific autoantigens presented by antigen-presenting cells induce cytotoxic T cell responses, resulting in β-cell damage and insulin deficiency [109,110]; moreover, the beneficial effects of vitamin D in T1DM are related to its impact on diverse types of immune cells [6]. Most immune cell populations, such as macrophages, dendritic cells, and activated T and B lymphocytes, express VDR [111,112,113]. Through VDR, 1,25(OH)_2_D inhibits T cell activation by downregulating the expression of major histocompatibility complex class II and co-stimulatory molecules, such as cluster of differentiation (CD) 40, CD80, and CD86, in macrophages [114]. In dendritic cells, 1,25(OH)_2_D downregulates co-stimulatory molecule expression, producing tolerogenic dendritic cells, which prevent autoimmune responses by inducing Treg development [115,116]. Furthermore, 1,25(OH)_2_D suppresses T cell infiltration and drives a shift from helper T cell (Th) 1 or Th17 to Th2, thereby preventing β-cell damage and T1DM initiation by reducing the secretion of multiple cytokines and chemokines [92,117,118,119,120]. In addition, 1,25(OH)_2_D also inhibits B-cell proliferation and maturation into memory B-cells [121].

Vitamin D augments insulin synthesis and its secretion from pancreatic β-cells [122,123,124]. Pancreatic β-cells express VDR, and the vitamin D response element presents in the human insulin receptor gene promoter region [125]. Therefore, the binding of 1,25(OH)_2_D to VDR upregulates genes related to β-cell growth, insulin synthesis, and glucose transport [126,127]. Vitamin D also depolarizes cytoplasmic membranes and opens calcium channels in β-cells, thus increasing intracellular calcium levels and triggering insulin release by promoting insulin vesicle exocytosis [128,129,130].

### 4.2. In Vitro and In Vivo Studies

Numerous in vitro studies have demonstrated that vitamin D signaling is involved in islet dysfunction (Table 3). Vitamin D deficiency reportedly impairs islet insulin secretion, whereas vitamin D supplementation restores it [123,124,131,132]. Under pro-inflammatory cytokine treatment, 1,25(OH)_2_D has been found to upregulate anti-apoptotic proteins, thereby decreasing the apoptosis of RINm5F rat β-cells, MIN-6 mouse insulinoma cells, and isolated human islets [133,134,135]. Moreover, 1,25(OH)_2_D treatment has also been observed to upregulate transmembrane protein 27, an inducer of β-cell proliferation, in INS-1 cells [136,137]. Vitamin D analogs, such as calcipotriol, MC903, and KH1060, reportedly protect human islets from cytokine-induced cell death and improve insulin release from rat islets [138,139]. On the one hand, VDR knockdown was found to decrease Ins2 (insulin-encoding gene) expression and insulin secretion in mice [140] as well as increase cytokine-induced cell death in INS-1 rat insulinoma cells and human-induced pluripotent stem cells differentiated into pancreatic β-cell-like cells [138]. On the other hand, VDR overexpression was able to rescue islet dysfunction [141].

Vitamin D signaling has also been shown to prevent the initiation and progression of T1DM in multiple animal models (Table 3). Although a vitamin-D-deficient diet reportedly decreased insulin secretion in rats [123,154,155], 1,25(OH)_2_D or vitamin D analog treatment suppressed the incidence of hyperglycemia and insulitis in NOD mice [117,145,146]. In rodents injected with streptozotocin (STZ; a diabetogenic chemical), vitamin D significantly improved pancreatic β-cell function and increased blood glucose, insulin, and HbA1c levels [134,147,148]. Vitamin D and its analogs, including Ro26-2198, improved pancreatic β-cell survival and insulin content in NOD and T1DM mice [91,117,149]. Another vitamin D analog, 2α-methyl-19-nor(20S)-1,25(OH)_2_D, decreased T1DM progression in Ins2-lacking mice [152]. Mice with VDR overexpression reportedly exhibited STZ resistance, β-cell mass preservation, and elevated β-cell proliferation marker expression [141].

## 5. Role of Vitamin D in Insulin Signaling and Insulin Resistance

Serum vitamin D concentration is linked to metabolic health. Chronic and systemic inflammation can cause metabolic dysfunction, including insulin resistance, and vitamin D suppresses pro-inflammatory responses as an anti-inflammatory hormone [6,156,157].

### 5.1. Mechanisms

Inflammation impairs insulin signaling, and vitamin D suppresses inflammatory responses by directly inhibiting nuclear factor-kappa B (NF-κB) activation and endoplasmic reticulum (ER) stress. Via VDR, vitamin D downregulates NF-ĸB target genes, including toll-like receptors (TLR2 and TLR4), pro-inflammatory cytokines (IL-1β, IL-6, and TNF-α), and chemokines (CCL2, CCL5, CXCL10, and CXCL11) [158,159,160,161], and ER stress inducers, including phosphorylated protein kinase R-like ER kinase, phosphorylated inositol-requiring enzyme 1 alpha, and CCAAT-enhancer-binding protein homologous protein [162].

Vitamin D also inhibits macrophage infiltration and polarizes macrophages to anti-inflammatory M2 states in adipose tissues [163]. Mature adipocytes are insulin sensitive, and inflammation-induced adipocyte dedifferentiation causes insulin resistance [164]. Vitamin D promotes adipose differentiation by upregulating adipogenic regulators, such as peroxisome proliferator-activated receptor gamma (PPARγ) and CCAAT-enhancer-binding protein alpha, and functional proteins, including fatty-acid-binding protein 4 and lipoprotein lipase (LPL) [165,166,167,168].

Other mechanisms may involve the protective effects of vitamin D against insulin resistance. In vitamin D deficiency, vitamin D treatment increases the secretion of parathyroid hormone, which upregulates glucose transporter (GLUT) 1 and GLUT4 expression and mitigates insulin resistance [169,170]. Vitamin D inhibits the renin–angiotensin–aldosterone system, which impairs β-cell function and GLUT4 recruitment [171]. Vitamin D supplementation also activates the 5’ adenosine monophosphate-activated protein kinase pathway and prevents ROS formation, thus alleviating insulin resistance [172].

### 5.2. In Vitro and In Vivo Studies

Vitamin D has been shown to regulate insulin sensitivity in multiple in vitro and in vivo models (Table 3). Through VDR, 1,25(OH)_2_D upregulated insulin receptor expression in U-937 human promonocyte-like cells [126,142] and increased glucose uptake via insulin receptor substrate 1 phosphorylation at the tyrosine residue in C2C12 myotubes and 3T3-L1 adipocytes [143,144]. In rat epitrochlearis muscles, VDR activation enhanced GLUT4 translocation by increasing intracellular calcium concentrations [173]. Although VDR deletion promoted insulin resistance in mouse liver [174], VDR activation reduced hepatic inflammation and insulin resistance in diet-induced obese mice [153]. Treatment with 1,25(OH)_2_D improved insulin sensitivity via PPARγ in ovariectomized rats [150] and reversed diabetes-induced downregulation of the insulin receptor in STZ-treated diabetic rats [151].

## 6. Conclusions

**Figure 2:**
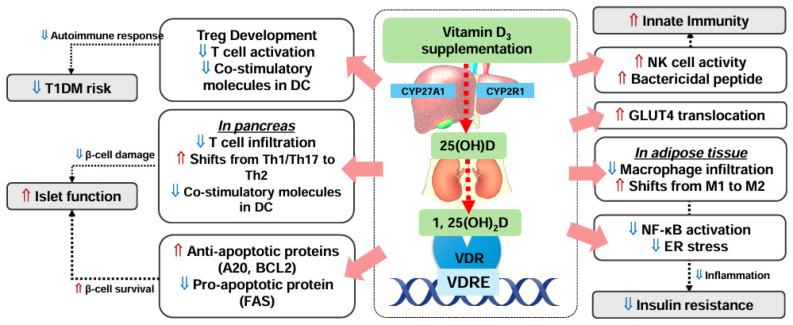
Multifaceted roles of vitamin D for diabetes. Abbreviations: ⇑, increase; ⇓, decrease; 25(OH)D, 25-hydroxyvitamin D; 1,25(OH)_2_D, 1,25-dihydroxyvitamin D; VDR, vitamin D receptor; VDRE, vitamin D response element; T1DM, type 1 diabetes mellitus; Treg, regulatory T cell; DC, dendritic cell; Th, helper T cell; A20, also known as TNF-α-induced protein 3; BCL2, BCL2 apoptosis regulator; FAS, Fas cell surface death receptor; NK, natural killer; GLUT, glucose transporter; NF-κB, nuclear factor-kappa B; ER, endoplasmic reticulum.

A close relationship between vitamin D status and diabetes has been reported in both cross-sectional studies and RCTs. Vitamin D’s innate immunity-enhancing effect can provide protection against infections in people with diabetes, who are particularly vulnerable. Additionally, the anti-inflammatory effects of vitamin D can mitigate complications associated with diabetes and islet dysfunction. Figure 2 depicts the roles of vitamin D for diabetes. Maintaining adequate vitamin D status has become important for several reasons including the global prevalence of vitamin D deficiency or insufficiency, the increasing number of individuals diagnosed with T2DM who are younger than 40 years old, longer life expectancy, leading to a higher number of people living with diabetes, and rising obesity rates. Vitamin D supplementation has shown mixed outcomes in the prevention of T2DM; however, more consistent benefits have been observed in reducing the risk of T1DM. Additionally, vitamin D supplementation has demonstrated a consistent beneficial effect in improving insulin resistance among persons with T2DM.

## Figures and Tables

**Table 1:** Vitamin D status and diabetes.

**Vitamin D Status and Type 2 Diabetes**			
**Study Design**	**Characteristics of Participants**	**Outcome**	**Reference**
Cross-sectional study	3rd NHANES, 6228 US population, >20 y	Lower OR for diabetes in highest vitamin D quartile in Mexican Americans and non-Hispanic Whites	(Scragg, Sowers, and Bell 2004) [8]
Cross-sectional study	3rd NHANES, 12,719 US population without diabetes, >20 y	Lower serum 25(OH)D levels were associated with prediabetes after adjustment	(Shankar, Sabanayagam, and Kalidindi 2011) [9]
Cross-sectional study	9014 Korean adults, >50 y,	Lower OR for T2DM in insufficient (20 to 30 ng/mL) or sufficient (>30 ng/mL) vitamin D group	(Nam et al. 2017) [10]
Cross-sectional study	480 Japanese population (35–79 y)	Higher OR for T2DM in lowest vitamin D quartile	(Nakamura et al. 2023) [11]
Cross-sectional study	150 healthy glucose-tolerant subjects	Plasma 25(OH)D levels positively associated with β-cell function and insulin sensitivity	(Karnchanasorn, Ou, and Chiu 2012) [16]
Cross-sectional study	151 Kenyan T2DM patients (mean age: 58.2y)	Blood vitamin D level inversely correlated with glycemic control and BMI	(Karau et al. 2019) [14]
Case–control study	2659 from the Henan Rural Cohort (NGT: 897, IFG: 913, T2DM: 849)	Lower OR for IFG in highest vitamin D quartile (Q4 vs. Q1) and lower OR for T2DM in Q2 and Q3 (vs. Q1)	(Wang et al. 2020) [13]
(1) Cross-sectional study (2) Prospective cohort study	The Irish Longitudinal Study on Ageing (TILDA): 5272 (wave 1) and 3838 (wave 2), ≥50 y, 4 y follow-up	(1) Increased RRR of having prevalent diabetes was associated with baseline 25(OH)D level (2) 62% increased likelihood of developing prediabetes in vitamin-D-deficient (<12 ng/mL) compared to vitamin-D-insufficient (>30 ng/mL) cases	(McCarthy et al. 2022) [12]
Prospective cohort study	9814 from general population (810 developed T2DM), 29 y follow-up	Lower 25(OH)D concentrations were associated with higher cumulative incidence of T2DM	(Afzal, Bojesen, and Nordestgaard 2013) [17]
Prospective cohort study	Randomly selected 524 non-diabetic population (40–69 y), 10 y follow-up	Baseline serum 25(OH)D inversely associated with 10 y risk of hyperglycemia	(Forouhi et al. 2008) [19]
Nested case–control study	7503 Finnish population (412 developed T2DM), 22 y follow-up	Higher baseline 25(OH)D reduced risk of T2DM	(Knekt et al. 2008) [18]
Nested case–control study	5140 women (mean age: 66 y; 317 developed T2DM), 7.3 y follow-up	Baseline 25(OH)D was not associated with T2DM incidence	(Robinson et al. 2011) [20]
Prospective cohort study	351 aged participants (51% females; 67.9 ± 5.7 years; 45 developed diabetes), 7–9 y follow up	No significant association between 25(OH)D and diabetes incidence, but a significant association with follow-up HbA1c levels	(Pilz et al. 2012) [21]
**Vitamin D Status and Type 1 Diabetes**			
**Study Design**	**Characteristics of Participants**	**Outcome**	**Reference**
Case–control study	Qatari population (<16 y) 170 case and 170 control	Vitamin D deficiency was higher in T1DM children than in control	(Bener et al. 2009) [25]
Case–control study	88 newly diagnosed T1DM and 57 control (mean age: 14.6 y)	Blood 25(OH)D and 1,25(OH)_2_D are lower in T1DM case	(Pozzilli et al. 2005) [26]
(1) Prospective study (2) Cross-sectional study	(1) 108 children (with multiple islet auto Ab), median 5.8 y follow-up (2) 406 (negative), 244 (newly T1DM)	(1) Incidence of T1DM is not different between vitamin-D-deficient and -sufficient populations (2) Blood 25(OH)D was lower, and prevalence of vitamin D deficiency was higher in cases with multiple islet auto Ab than in those without	(Raab et al. 2014) [34]
Cross-sectional study	83 Finnish (29 diabetes-associated auto Ab positive), 32 Estonian (6 positive) children	Vitamin D status was not different between diabetes-associated auto Ab positivity	(Reinert-Hartwall et al. 2014) [35]
Prospective study	1316 youth with islet-auto-Ab-positive T1DM, mean follow-up 24.3 mo	Baseline 25(OH)D inversely associated with fasting C-peptide	(Mayer-Davis et al. 2013) [36]
Multicenter study	459 new T1DM (15–34 y) cases and 208 control subjects, 8 y follow-up	Baseline 25(OH)D is lower in T1DM, and, 8 y later, 25(OH)D decreased in T1DM cases	(Littorin et al. 2006) [27]
Case–control study	56 T1DM, 46 control children	Blood 25(OH)D is lower in T1DM cases No difference in VDR polymorphism	(Greer et al. 2013) [28]
Case–control study	82 T1DM, 117 control children	T1DM children showed higher 25(OH)D deficiency	(Federico et al. 2018) [29]

Ab, antibody; IFG, impaired fasting glucose; mo, month; NGT, normal glucose tolerance; NHANES, National Health and Nutrition Examination Survey; OR, odds ratio; RRR, Relative Risk Ratio; T1DM, type 1 diabetes mellitus; T2DM, type 2 diabetes mellitus; y, year.

**Table 2:** Preventive or therapeutic effects of vitamin D supplementation on diabetes.

**Preventive Effect of Vitamin D on Type 2 Diabetes**				
**Study Design**	**Characteristics of Participants**	**Treatment (Dose and Duration)**	**Outcome**	**Reference**
RCT	2423 with pre-DM	4000 IU/day vitamin D_3_ or placebo, 2.5 y	No difference in hazard ratio of T2DM between Suppl and placebo	(Pittas et al. 2019) [41]
RCT	511 with pre-DM (mean age: 62 y, 314 males)	2000 IU/day vitamin D_3_ or placebo, 5 y	No difference in hazard ratio of T2DM between Suppl and placebo	(Jorde et al. 2016) [42]
RCT	1256 with pre-DM (>30 y)	0.75 μg/day eldecalcitriol or placebo, 3 y	No difference in hazard ratio of T2DM between Suppl and placebo	(Kawahara et al. 2022) [44]
RCT	92 with pre-DM	2000 IU/day vitamin D_3_ or placebo, 16 wk	↑ Pancreatic β-cell function in Suppl	(Mitri et al. 2011) [45]
RCT	96 with pre-DM	5000 IU/day vitamin D_3_ or placebo, 6 mo	↑ Pancreatic β-cell function and insulin sensitivity in Suppl	(Lemieux et al. 2019) [46]
RCT	89 with pre-DM or new T2DM	4000 IU/day vitamin D_3_ or placebo, 12 wk	↑ Insulin secretion and sensitivity in Suppl No difference in disposition index or glycemia	(Harris, Pittas, and Palermo 2012) [47]
RCT	130 with pre-DM	1200 IU/day vitamin D_3_ or placebo, 16 wk	No difference in insulin sensitivity Insulinogenic index ↑ β-cell function increased in Suppl	(Oosterwerff et al. 2014) [48]
**Therapeutic Effect of Vitamin D on Type 2 Diabetes**				
	**Characteristics of Participants**	**Treatment (Dose and Duration)**	**Outcome**	**Reference**
Meta-analysis of RCT	46 RCTs (2164 intervention, 2149 placebo) patients with T2DM (>18 y)	Oral (42 studies) or intramuscular injection (4 studies) vitamin D supplementation for 8–48 wk supplementation duration	Vitamin D supplementation resulted in a reduction in FPG, HbA1c, and HOMA-IR	(Oosterwerff et al. 2014; Farahmand et al. 2023) [48,49]
Meta-analysis of RCT	20 clinical trials involving 2703 adults with T2DM (48–67 y)	Oral vitamin D supplementation for 2–6 mo of vitamin D3 (17 studies) and vitamin D2 (1 study) supplementation	HOMA-IR improved in Suppl, not in other outcomes Subgroup analysis of non-obese, Middle Eastern, large-dose, short-term, baseline-vitamin-D-deficient population: ↓ insulin resistance	(Li et al. 2018) [50]
Meta-analysis of RCT	(1) 18 RCTs involving 1243 with T2DM (2) 20 observational studies (11,063 participants)	(1) Oral vitamin D supplementation for 8–24 wk	(1) Vitamin D supplementation improved insulin, glucose, HOMA-IR (2) Vitamin D status inversely correlated with insulin, glucose, HOMA-IR	(Lei et al. 2023) [51]
**Preventive Effect of Vitamin D on Type 1 Diabetes**				
**Study Design**	**Characteristics of Participants**	**Treatment (Dose and Duration)**	**Outcome**	**Reference**
Multicenter study	Pregnant women (offspring: 85 T1DM, 1071 control)	Cod liver oil supplementation during pregnancy	Cod liver oil supplementation of mothers: ↓ the OR of T1DM of offspring	(Stene et al. 2000) [52]
Birth cohort study	12,055 pregnant women (10,821 children followed up at age 1 y, 81 T1DM)	Various amounts of vitamin D supplementation (questionnaire)	Vitamin D supplementation in first year: ↓ frequency of T1DM	(Hyppönen et al. 2001) [53]
Case–control study	820 T1DM, 2335 controls	Various amounts of vitamin D supplementation (questionnaire)	Vitamin D supplementation in early childhood: ↓ the risk of T1DM	(Group 1999) [54]
Birth cohort study	233 mothers with high risk of T1DM (children 0.8–7.3 y follow-up)	Questionnaire on vitamin D supplementation	Maternal intake of vitamin D supplementation: ↓ the risk of islet autoimmunity	(Fronczak et al. 2003) [55]
**Therapeutic Effect of Vitamin D on Type 1 Diabetes**				
**Study Design**	**Status of Participants**	**Treatment (Dose and Duration)**	**Outcome**	**Reference**
RCT	70 with newly diagnosed T1DM	0.25 μg 1,25(OH)_2_D/2 day or 25 mg/kg/d nicotinamide, 1 y	↑ Fasting C-peptide and ↓ daily insulin dose in the calcitriol group No difference in baseline/stimulated C-peptide or HbA1c between calcitriol and nicotinamide group	(Pitocco et al. 2006) [56]
RCT	38 with newly diagnosed T1DM (7–30 y)	2000 IU/d of vitamin D_3_ for 18 mo	↑ CCL2, Treg% ↓ Cumulative incidence of undetectable fasting C-peptide in Suppl compared with placebo	(Gabbay et al. 2012) [57]
RCT	29 with newly diagnosed T1DM	70 IU/kg/day vitamin D_3_ for 12 mo	↑ Suppressive capacity of Tregs in Suppl from baseline to 3, 6, and 12 mo and higher than placebo at 12 mo	(Treiber et al. 2015) [58]
RCT	34 newly diagnosed T1DM (11–35 y)	0.25 μg 1,25(OH)_2_D/d for 2 y	No difference in HbA1C and insulin requirement between Suppl and placebo at 6, 12, and 24 mo follow-up	(Bizzarri et al. 2010) [59]
RCT	(1) 25 with T1DM (2) 40 with T1DM and 18 placebo (18–39 y)	(1) 0.25 μg 1,25(OH)_2_D/d for 18 mo (2) 0.25 μg 1,25(OH)_2_D/d for 9 mo	No differences in AUC C-peptide, peak C-peptide, and fasting C-peptide between Suppl and placebo	(Walter et al. 2010) [60]
RCT	27 with new-onset T1DM (mean age: 22 y)	0.25 μg 1,25(OH)_2_D/day for 1 y	No difference in HbA1c and insulin requirement between Suppl and placebo	(Napoli et al. 2013) [61]
RCT	52 Indian children with T1DM	60,000–120,000 IU/mo, vitamin D for 6 mo	↑ C-peptide in Suppl No difference in HbA1c and insulin requirement	(Sharma et al. 2017) [62]
RCT	44 children with T1DM	300,000 IU vitamin D_3_ single-dose intramuscular injection with Ca (40 mg/kg/d) for 3 mo	↓ HbA1c in Suppl	(Mohammadian et al. 2015) [63]

↑, increase; ↓, decrease; AUC, area under curve; CCL2, C-C motif chemokine ligand 2; d, day; FPG, fasting plasma glucose; HbA1c, glycated hemoglobin; HOMA-IR, homeostasis model assessment of insulin resistance; mo, month; OR, odds ratio; RCT, randomized controlled trial; T1DM, type 1 diabetes mellitus; T2DM, type 2 diabetes mellitus; Suppl, vitamin D supplementation group; placebo: placebo group; wk, week; y, year.

**Table 3:** In vitro and in vivo effects of vitamin D or vitamin D analog on diabetes.

Experimental Model	Treatment (Dose and Duration)	Outcome	Reference
RINm5F rat β-cells	1,25(OH)_2_D (10 nM or 1 μM; 48 h)	↑ A20 (anti-apoptotic protein) expression under cytokine mixture treatment	(Riachy et al. 2002) [133]
MIN-6 mouse insulinoma cells	1,25(OH)_2_D (0.01 nM; 24 h)	↑ BCL2 (anti-apoptotic protein) expression under STZ treatment	(Wang et al. 2016) [134]
Human pancreatic islets	1,25(OH)_2_D (10 nM or 1 μM; 48 h)	↓ FAS (pro-apoptotic protein) expression under cytokine mixture treatment	(Riachy et al. 2006) [135]
INS-1 rat insulinoma cells	1,25(OH)_2_D (10 nM; 24 h or 48 h)	↑ TMEM27 (an inducer of β-cell proliferation) expression	(Pepaj et al. 2014) [136]
U-937 human promonocyte-like cells	1,25(OH)_2_D (1–100 nM; 24 h)	↑ IR mRNA expression	(Maestro et al. 2000) [142]
U-937 human promonocyte-like cells	1,25(OH)_2_D (0.01–100 nM; 24 h)	↑ Insulin-induced glucose oxidation	(Maestro et al. 2002) [126]
C2C12 myotubes	1,25(OH)_2_D (10 nM; 48 h)	↑ IRS1 phosphorylation at tyrosine residue	(Zhou et al. 2008) [143]
3T3-L1 adipocytes	1,25(OH)_2_D (25 or 50 nM; 2 h)	↑ IRS1 phosphorylation at tyrosine residue	(Manna, Achari, and Jain 2017) [144]
Human pancreatic islets	Calcipotriol (100 nM; 1 or 4 wk)	↑ Ex vivo islet survival and insulin secretion	(Wei et al. 2018) [138]
Rat pancreatic islets	MC903 or KH1060 (0.1 nM; 28–72 h)	↑ Insulin secretion under IL-1β treatment	(Sandler, Buschard, and Bendtzen 1994) [139]
NOD mice	1,25(OH)_2_D (5 μg/kg per 2 d; 100 d)	↓ Incidence of insulitis	(Mathieu et al. 1992) [145]
NOD mice	1,25(OH)_2_D (5 μg/kg per 2 d; 200 d)	↓ Incidence of glucosuria and hyperglycemia	(Mathieu et al. 1994) [146]
STZ-injected CD-1 mice	1α(OH)D (0.3 μg/kg/d; 1 mo)	↓ Incidence of hyperglycemia and insulitis	(Inaba et al. 1992) [147]
STZ-injected Wistar rats	Vitamin D (10 IU/kg/d; 2 mo)	↑ Pancreatic β-cell function ↓ Blood glucose and HbA1c levels	(Sadek and Shaheen 2014) [148]
STZ-injected C57BL/6J mice	1,25(OH)_2_D (5 μg/kg per 2 d; 3 wk)	↓ Incidence of diabetes	(Wang et al. 2016) [134]
NOD mice	1,25(OH)_2_D (5 μg/kg per 2 d; 4, 8, or 12 wk)	↑ Insulin content in pancreas ↓ Insulitis score	(Gysemans et al. 2005) [149]
T1D mice	Vitamin D (2200 IU/kg/d; 4 wk)	↑ Pancreatic β-cell survival and insulin content	(Lai et al. 2022) [91]
Ovariectomized rats	Vitamin D (100, 1000, 10,000 IU/kg/wk; 8 wk)	↑ Insulin sensitivity through PPARγ activation	(Hoseini, Damirchi, and Babaei 2017) [150]
STZ-injected Wistar rats	Vitamin D_3_ (12 μg/kg/d; 15 d)	↑ IR mRNA expression	(George et al. 2012) [151]
NOD mice	Ro 26-2198 (0.03 μg/kg, 5 times/wk; 1 or 2 mo)	↓ Incidence of hyperglycemia and insulitis	(Gregori et al. 2002) [117]
*Ins2*-lacking NOD mice	2α-methyl-19-nor(20S)-1,25(OH)_2_D (600 pg/d; 3 mo)	↓ Insulitis score and T1D progression	(Kiekhaefer et al. 2011) [152]
Diet-induced obese mice	Calcipotriol (20 μg/kg/d; 4 wk)	↓ Hepatic insulin resistance and inflammation	(Dong et al. 2020) [153]

↑, increase; ↓, decrease; A20, also known as TNF-α-induced protein 3; BCL2, BCL2 apoptosis regulator; d, day; FAS, Fas cell surface death receptor; h, hour; HbA1c, glycated hemoglobin; IL-1β, interleukin-1beta; *Ins2*, insulin-encoding gene; IR, insulin receptor; IRS1, insulin receptor substrate 1; mo, month; NOD, non-obese diabetic; PPARγ, peroxisome proliferator-activated receptor gamma; STZ, streptozotocin; T1D, type 1 diabetes; TMEM27, transmembrane protein 27.
